# Immune-related gene risk score predicting the effect of immunotherapy and prognosis in bladder cancer patients

**DOI:** 10.3389/fgene.2022.1011390

**Published:** 2022-10-04

**Authors:** Yuantao Zou, Gangjun Yuan, Xingliang Tan, Sihao Luo, Cong Yang, Yi Tang, Yanjun Wang, Kai Yao

**Affiliations:** ^1^ Department of Urology, Sun Yat-sen University Cancer Center, Guangzhou, China; ^2^ State Key Laboratory of Oncology in Southern China, Guangzhou, China; ^3^ Collaborative Innovation Center of Cancer Medicine, Guangzhou, China; ^4^ Department of Urology Oncological Surgery, Chongqing University Cancer Hospital, Chongqing, China

**Keywords:** IRGRS, bladder cancer, immunotherapy, microenvironemnt, prognosis

## Abstract

**Background:** Immune checkpoint inhibitor therapy has changed the treatment model of metastatic bladder cancer. However, only approximately 20% of patients benefit from this therapy, and robust biomarkers to predict the effect of immunotherapy are still lacking. In this study, we aimed to investigate whether immune-related genes could be indicators for the prognosis of bladder cancer patients and the effect of immunotherapy.

**Methods:** Based on bladder cancer dataset from the Cancer Genome Atlas (TCGA) and GSE48075, 22 immune microenvironment-related cells were identified by CIBERSORT. After performing a series of bioinformatic and machine learning approaches, we identified distinct tumor microenvironment clusters and three bladder cancer specific immune-related genes (EGFR, OAS1 and MST1R). Then, we constructed immune-related gene risk score (IRGRS) by using the Cox regression method and validated it with the IMvigor210 dataset.

**Results:** IRGRS-high patients had a worse overall survival than IRGRS-low patients, which was consistent with the result in the IMvigor210 dataset. Comprehensive analysis shows that patients with high IRGRS scores are mainly enriched in basal/squamous type (Ba/Sq), and tumor metabolism-related pathways are more Active, with higher TP53 and RB1 gene mutation rates, lower CD4+/CD8+ T cell infiltration, higher M0 macrophage infiltration, and lower immunotherapy efficacy. In contrast, Patients with low IRGRS scores are mainly enriched in the luminal papillary type (LumP), which is associated with the activation of IL-17 and TNF signaling pathways, higher mutation rates of FGFR3 and CDKN1A genes, higher CD4+/CD8+ T cell infiltration content, and The level of M0 macrophage infiltration was relatively low, and the immunotherapy was more probably effective.

**Conclusion:** Our study constructed an IRGRS for bladder cancer and clarified the immune and molecular characteristics of IRGRS-defined subgroups of bladder cancer to investigate the association between IRGRS and its potential implications for prognosis and immunotherapy.

## Introduction

Bladder cancer (BLCA) is one of the most prevalent urinary tract malignancies, with an estimated 430,000 new cases and 165,000 deaths worldwide (Lenis et al., 2020). Immunotherapies such as anti-PD-1/PD-L1 inhibitors have demonstrated substantial antitumour activity in advanced and metastatic BLCA, although cisplatin-based chemotherapy and radical cystectomy are still the first-line treatments for muscle-invasive BLCA (Jordan and Meeks, 2019; Patel et al., 2020). However, patients with advanced or metastatic BLCA ineligible for cisplatin only showed an objective remission rate (ORR) of 23%, and the median OS was 15.9 months after receiving the PD-L1 inhibitor atezolizumab as treatment in a phase II trial (Balar et al., 2017). Although some advanced DNA methylation based urinary assay could detect the early stage bladder cancer leading to early treatment, the prognosis of bladder is still unsatisfied (Chen et al., 2020). Besides, how to screen out patients suitable for immunotherapy is still an urgent problem to be solved. At present, the standard biomarkers for clinicians to select patients who are eligible for immunotherapy are immunohistochemistry assays for PD-L1 protein and tumour mutation burden (TMB), but some studies have found conflicting results when using the two biomarkers to predict immunotherapy response or overall survival. Furthermore, many patients whose tumours have low or no detectable PD-L1 expression can also benefit from immunotherapy (Rosenberg et al., 2016). There was no significant association between high TMB and the efficacy of immunotherapy in BLCA (Necchi et al., 2018; Powles et al., 2019). Therefore, it is crucial to develop robust predictive biomarkers to predict the effect of immunotherapy and the prognosis of BLCA patients. Although there have been some studies on the development of molecular markers for the efficacy of immunotherapy (Zhang et al., 2021a; Cao et al., 2021), they did not elucidate the mechanisms behind the molecular markers.

In this study, we analysed three BLCA transcriptomic datasets from patient cohorts (GSE48075, TCGA-BLCA, and IMvigor210). We used the GSE48075 and TCGA-BLCA datasets as training sets to identify the hub genes related to the immune microenvironment. Two computational algorithms, namely, CIBERSORT and ESTIMATE, were used to analyse the expression levels of 22 immune cell types and cancer-related fibroblasts to profile the immune landscape of bladder cancer. Then, we divided patients into different subgroups and examined the correlations of the subgroups with corresponding genomic characteristics and clinical features. Finally, we constructed IRGRS based on the expression of three immune-related genes. The IRGRS was verified to be a robust prognostic biomarker to predict the response to immune checkpoint inhibitors and prognosis.

## Materials and methods

### Dataset and processing

The Bladder Cancer Dataset from TCGA was used in this study. BLCA samples (*n* = 412) with both RNA sequencing (RNA-seq) data and detailed follow-up information were included for further analysis. RNA-seq data of 270 bladder samples (GSE48075) and corresponding survival information were downloaded from the Gene Expression Omnibus (GEO). IMvigor210 was a cohort in which 195 muscle invasive bladder cancer (MIBC) patients were treated with an anti-PD-L1 agent (atezolizumab) to evaluate the effect of immunotherapy in locally advanced or metastatic urothelial bladder cancer (Mariathasan et al., 2018). Genome, transcriptomic, and clinical data can be downloaded from http://research-pub.gene.com/IMvigor210CoreBiologies. We removed samples whose survival data were not available and then carried out logarithmic processing by the “voom” function of the R package “Limma” (Ritchie et al., 2015). All the RNA-seq datasets in the form of fragments per kilobase of transcript per million mapped reads (FPKM) values were converted into transcripts per million (TPM) to make samples from TCGA and GEO more comparable.

### Inference of immune infiltrating cells in the tumour environment

To calculate the composition ratio of 22 tumour-infiltrating immune cells in each cancer sample, CIBERSORT was utilized based on the preset 547 barcode genes of the gene expression matrix (Newman et al., 2015). CIBERSORT is a deconvolution algorithm to estimate immune cell type (including B cells, T cells, natural killer cells, macrophages, DCs, and myeloid subsets) proportions in data from tumour tissues with mixed cell types.

### Unsupervised consensus clustering of 22 tumour-infiltrating immune cells

Unsupervised clustering methods were applied to identify distinct immune patterns and to classify tumour samples for further analysis based on 22 tumour-infiltrating immune cell expression matrices. The R package “ConsensusClusterPlus” was used to perform the above procedure, and 1000 rounds were repeated to guarantee the robustness of classification (Wilkerson and Hayes, 2010). A consensus heatmap was mapped for each sequence of cluster numbers (*k* = 2, 3, 4, 5, … … ), and a progression graph and corresponding cumulative distribution function (CDF) were generated to determine the optimal cluster number.

### Identification of differentially expressed genes associated with immune subtypes

We classified patients into four distinct immune patterns by unsupervised consensus clustering to identify immune-related genes. The R package “Limma” was utilized to determine DEGs among the 4 immune subtypes (Ritchie et al., 2015). The criterion for selecting significant DEGs was an adjusted *p* value < 0.01.

### Construction of immune-related genes score

DEGs among all immune clusters were identified, and a union set of genes was extracted. First, we adopted an unsupervised clustering method based on all DEGs to classify patients into several groups for deeper analysis. Then, we defined the optimal number of gene clusters to perform weighted gene coexpression network analysis (WGCNA) to select the related modules of the gene cluster (Langfelder and Horvath, 2008). The “WGCNA” package in R software was used to construct an adjacency matrix with a soft threshold of *β* = 5, which was then transformed into a topological overlap matrix (TOM). The corresponding dissimilarity (1-TOM) was calculated as the distance to cluster genes. Then, we built a dynamic pruning tree to identify the related modules. Five modules were identified after setting the merging threshold function at 0.25. Gene significance (GS) and module membership (MM) were calculated for intramodular analysis to select the hub genes. GS is an absolute value to quantify the correlation between a specific gene and its phenotypic trait. MM shows the correlation between the gene and a given module. Hub genes were screened out by setting the cut-off criteria of GS > 0.01 and MM > 0.01. Then, we conducted K–M survival analysis to choose the genes associated with overall survival based on the expression value and clinical data of the hub gene. Then, a univariate Cox regression model was used to perform the prognostic analysis for genes selected after survival analysis. We utilized the least absolute shrinkage and selection operator (LASSO) to precisely predict the outcome of hub genes in BLCA patients. The IRGRS was then constructed by using the coefficients obtained by the LASSO–Cox algorithm, and the IRGRS was calculated by the sum of all gene expression levels multiplied by their corresponding coefficients.

### Immune characteristics and molecular biological differences between the high-IRGRS and low-IRGRS groups

According to the median value of the IRGRS in the training dataset, we separated the samples into two groups: the high IRGRS group and the low IRGRS group. To elucidate the underlying biological mechanism in different IRGRS groups, we used gene set enrichment analysis (GSEA), gene ontology (GO), and the Kyoto Encyclopedia of Genes and Genomes (KEGG) method with the clusterProfiler package of R (*p* < 0.05 and FDR<0.25) (Yu et al., 2012). Then we performed single sample GSEA (ssGSEA) analysis on several representative gene sets with the GSVA (Gene Set Variation Analysis) package of R (Hänzelmann et al., 2013). In addition, to further identify the differences in biological pathways between the high-IRGRS and low-IRGRS groups, GSVA enrichment analysis was conducted by using the “GSVA” package. GSVA is a method based on a nonparametric and unsupervised method to estimate the variation in pathway and biological process activity in the samples. We downloaded the gene sets of “c2.cp. kegg.v6.2.symbols” from the MsigDB database for GSVA. An adjusted *p* value less than 0.05 was regarded as statistically significant.

### Statistical analysis

The statistical significance of the mean value of variables between two groups was estimated by unpaired Student’s t tests. Correlation coefficients were computed using Spearman’s and distance correlation analyses. Spearman and distance correlation analyses were used to compute the correlation coefficients between each kind of TME infiltrating immune cell. Difference comparisons of three or more groups were conducted by one-way ANOVA and Kruskal–Wallis tests (Robertson et al., 2017). To determine the correlation between the IRGRS and patient survival, we divided patients into high- and low-IRGRS groups based on the median IRGRS value in the training group. The Kaplan-Meier method and log-rank tests were utilized to identify the significance of differences in the survival curves for the prognostic analysis. A univariate Cox regression model was adopted to compute the hazard ratios (HRs) in the process of selecting the hub genes. A multivariable Cox regression model was constructed to ascertain the independent prognostic factors. We assessed the specificity and sensitivity of the IRGRS by receiver operating characteristic (ROC) curve analysis and quantified the area under the curve (AUC) with the time ROC package. We used the maftools package to present the mutation landscape in patients with high and low IRGRS subtypes in the TCGA-BLCA cohort. All statistical *p* values were two-sided, with *p* < 0.05 indicating statistical significance. All data processing was done with R 4.0.2 software.

## Results

### Landscape of the tumour microenvironment of BLCA

**FIGURE 1:**
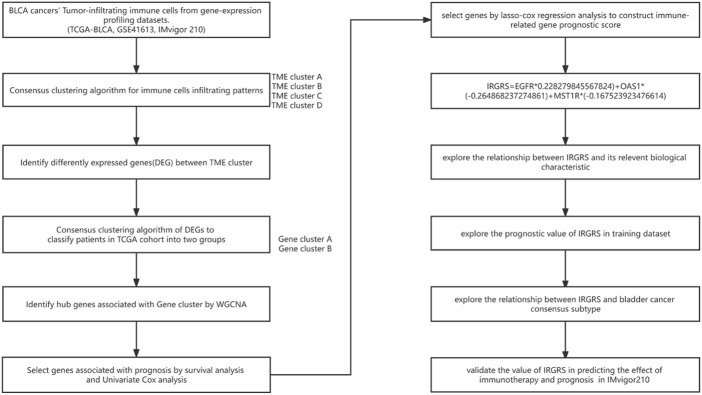
Overview of workflow about the study design.

**FIGURE 2:**
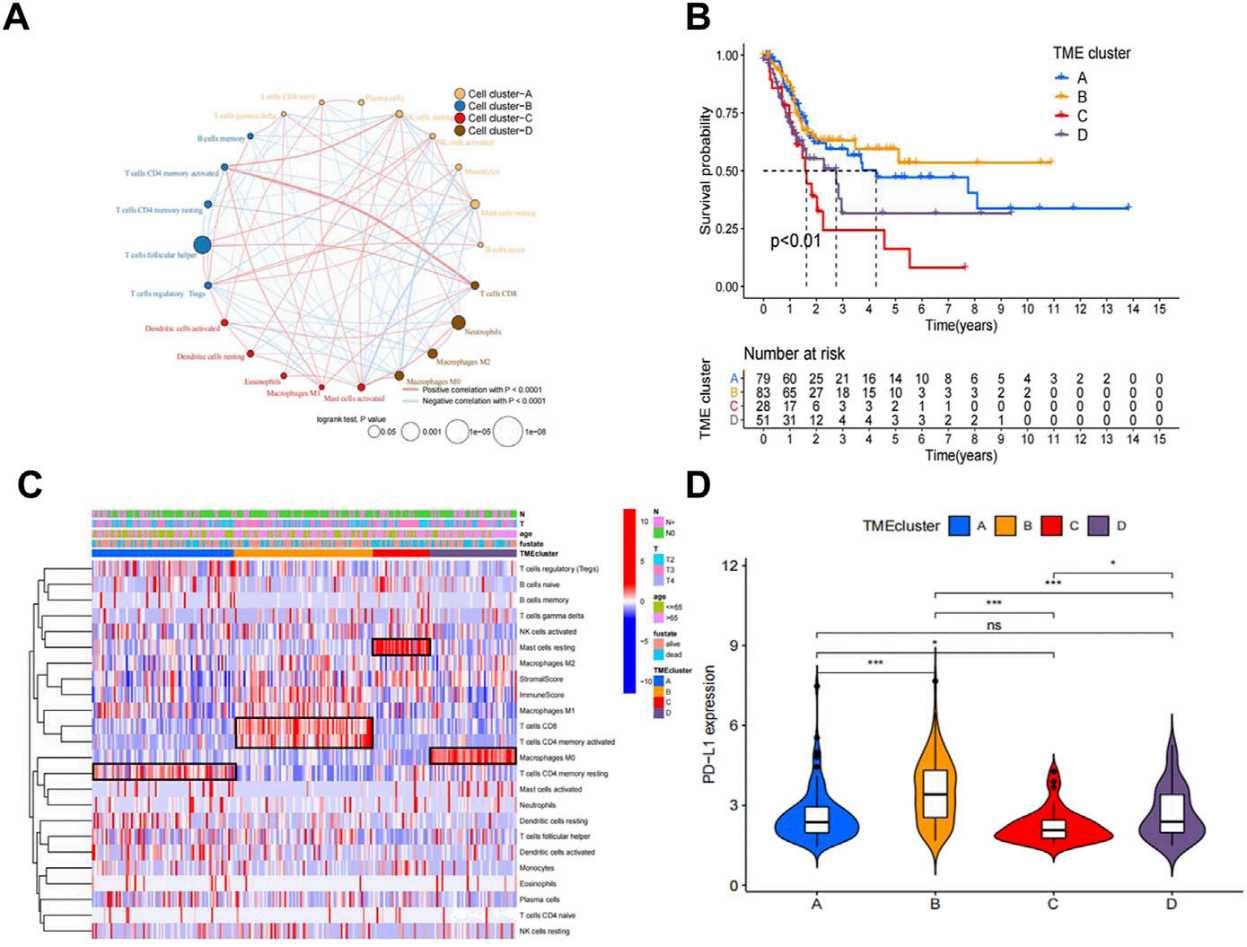
Landscape of the TME in bladder cancer and characteristics of TME subtypes. **(A)** The interaction between TME cell type in bladder cancer. Cell cluster A, orange; cell cluster B, blue; cell cluster C, red; cell cluster D, brown.The size of each cell represents survival impact of each TME cell type, calculation used the formula log10 (log-rank test *p* values indicated) respectively. The lines linking TME cells showed their interactions, and thickness represented the correlation estimated by Spearman correlation analysis and strength between regulators. Negative correlation was indicated with blue and positive correlation with red. **(B)**. Kaplan–Meier curves for overall survival (OS) of 241 bladder cancer patients from training cohorts (TCGA + GSE48075) with the TME infifiltration clusters. The numbers of patients in TMEcluster-A, -B, -C,and-D phenotypes are *n* = 79, *n* = 83, *n* = 28, *n* = 51 respectively. Log-rank test shows overall *p* < 0.01. **(C)**. Heatmap of 22 TME cells and ImmuneScore for 249 patients in the training cohort. **(D)**. Comparison of the PD-L1 among four TME subtypes in the training cohort.*p < 0.05, **p < 0.01, ***p < 0.001.

The workflow of how we constructed TME cell-infiltrating patterns and the IRGRS was systematically evaluated (Figure 1). The R package “ConsensusClusterPlus” was used to classify patients with different immune microenvironment patterns based on the amount of 22 tumour-infiltrated immune cells, and four distinct patterns termed TME clusters A, B, C, and D were recognized as the optimal cluster number after we evaluated clustering stability (Supplementary Figure S1C). The 22 tumour-infiltrated immune cell networks portrayed a comprehensive landscape of interactions and their impacts on the overall survival of patients with bladder cancer (Figure 2A; Supplementary Table S1). TME cluster B revealed a particularly prominent survival advantage, and TME cluster C showed the worst prognosis compared with that of the other three TME clusters (log-rank test, *p* < 0.01; Figure 2B). Taken together, we can conclude that crosstalk plays roles among different immune cells in the process of classifying distinct patterns. Then, we visualized the immune microenvironment of the four subtypes in a heatmap (Figure 2C), from which we could see that TME cluster A was characterized by high expression of CD4 memory activated T cells. TME cluster B was characterized by high expression of CD8^+^ T cells and CD4^+^ memory activated T cells. TME cluster C was characterized by high expression of resting mast cells. TME cluster D was characterized by high expression of M0 macrophages. A violin plot (Figure 2D) showed that TME cluster B had significantly higher PD-L1 expression than that of the other three TME clusters, and TME cluster C had the lowest PD-L1 expression among the four TME clusters. Except for TME clusters A and D, there were significant differences in the expression of PD-L1 between any two other groups.

### Construction of the TME signature and functional annotation

**FIGURE 3:**
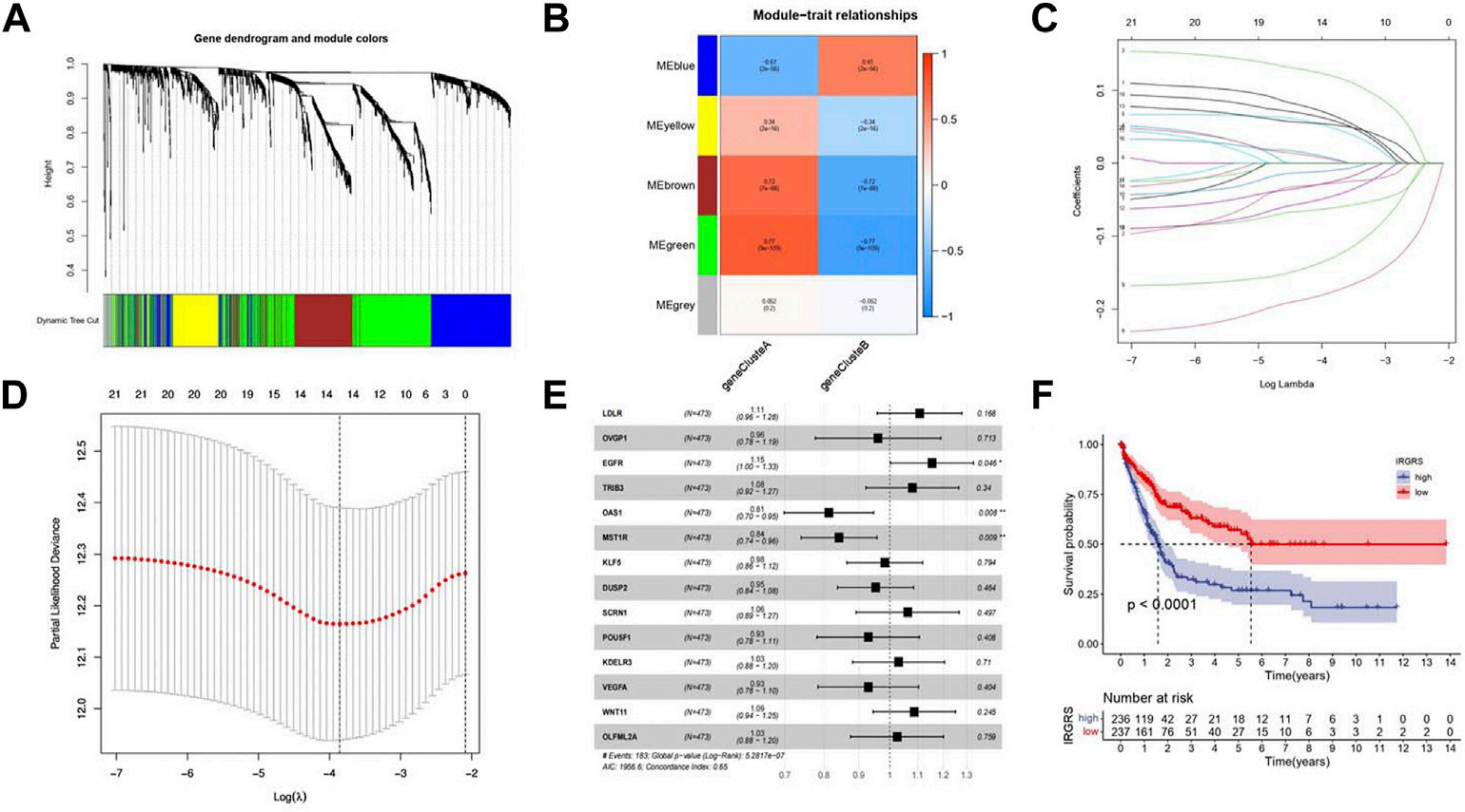
Methods about how to filter the hub genes to construct IRGRS system. **(A)**. Clustering dendrograms shows the relationshiop between gene and its corresponding module. **(B)**. Heatmap by WGCNA suggests Module-trait associations. Each row corresponds to a ME and column corresponds to genecluster. The number in the rectangle is the correlation coefficient, and the number in brackets is the corresponding *p* value. **(C)**. Least absolute shrinkage and selection operator (LASSO) coefficient profiles of 21 genes. **(D)**. Partial likelihood deviance for LASSO coefficient profiles. The vertical dotted line is shown at the optimal values, The red dots represent the partial likelihood values, the gray lines represent the standard error (SE). **(E)**. Forest plots of the multivariate Cox hazard model for overall survival. *p < 0.05, **p < 0.01, ***p < 0.001, ****p < 0.0001. Unadjusted HRs are shown with 95% confidence intervals. **(F)**. Survival analyses for high and low IRGRS patient groups in training cohort using Kaplan-Meier curves (*p* < 0.0001, Log-rank test).

To investigate the potential biological characteristics of each immune subtype, unsupervised analysis of DEGs gathered between each pattern was used to identify optimal genomic subtypes. Two gene clusters were recognized as the most suitable method to separate the training cohort population into 2 distant patient clusters (Supplementary Figure S2A), termed gene cluster A and gene cluster B. To obtain the gene cluster-related hub genes, WGCNA was carried out on all genes in gene clusters. Module membership (MM) is an index to measure the correlation between the gene and a given module (Langfelder and Horvath, 2008). Gene significance (GS) represents the correlation between the specific gene and gene cluster. Selected genes and their corresponding modules are shown in a heatmap (Figure 3A). We used a topological overlap matrix (TOM) to cluster all selected genes by dissimilarity measure based on the dynamic tree cut algorithm to divide the tree into five modules (Figure 3B) labelled with different colours. The results showed that the highest positive correlation coefficient between GS for the gene cluster and MM was in the green module (correlation coefficient = 0.77, *p* value = 9e-9), and the lowest negative correlation was in the blue module (correlation coefficient = −0.61, *p* value = 2e-56) (Figure 3B). The criteria for selecting the hub gene were MM > 0.01 and GS > 0.01. Among them, a total of 86 hub genes were identified in the green module (Supplementary Table S2), and 48 hub genes were identified in the blue module (Supplementary Table S3). To determine the independent prognostic genes, univariate Cox regression analysis and K-M survival analysis for OS were performed among the 134 hub genes in the blue and green modules. Twenty-one genes were determined by the selection criteria of Cox *p* value < 0.05 and K-M value < 0.05 (Supplementary Table S4). In order to solve the problem of overfitting of variables, we performed lasso-cox regression to remove 8 genes causing multicollinearity, and obtained 14 genes for subsequent analysis (Figures 3C,D). According to the results of the multivariate Cox hazard model, EGFR (*p* < 0.05), OAS1 (*p* < 0.01), and MST1R (*p* < 0.01) were significantly related to overall survival in BLCA patients (Figure 3E). Then, we constructed a prognostic index for all cancer samples calculated by the formula IRGRS = expression level of EGFR*0.228279845567824 + expression level of OAS1*(-0.264868237274861)+expression level of MST1R*(-0.167523923476614). We used the median IRGRS as the cut-off value, and high-IRGRS patients had a worse OS than low-IRGRS patients (*p* < 0.0001, log-rank test; Figure 3F).

### Molecular characteristics and functional enrichment analysis in different IRGRS subgroups

**FIGURE 4:**
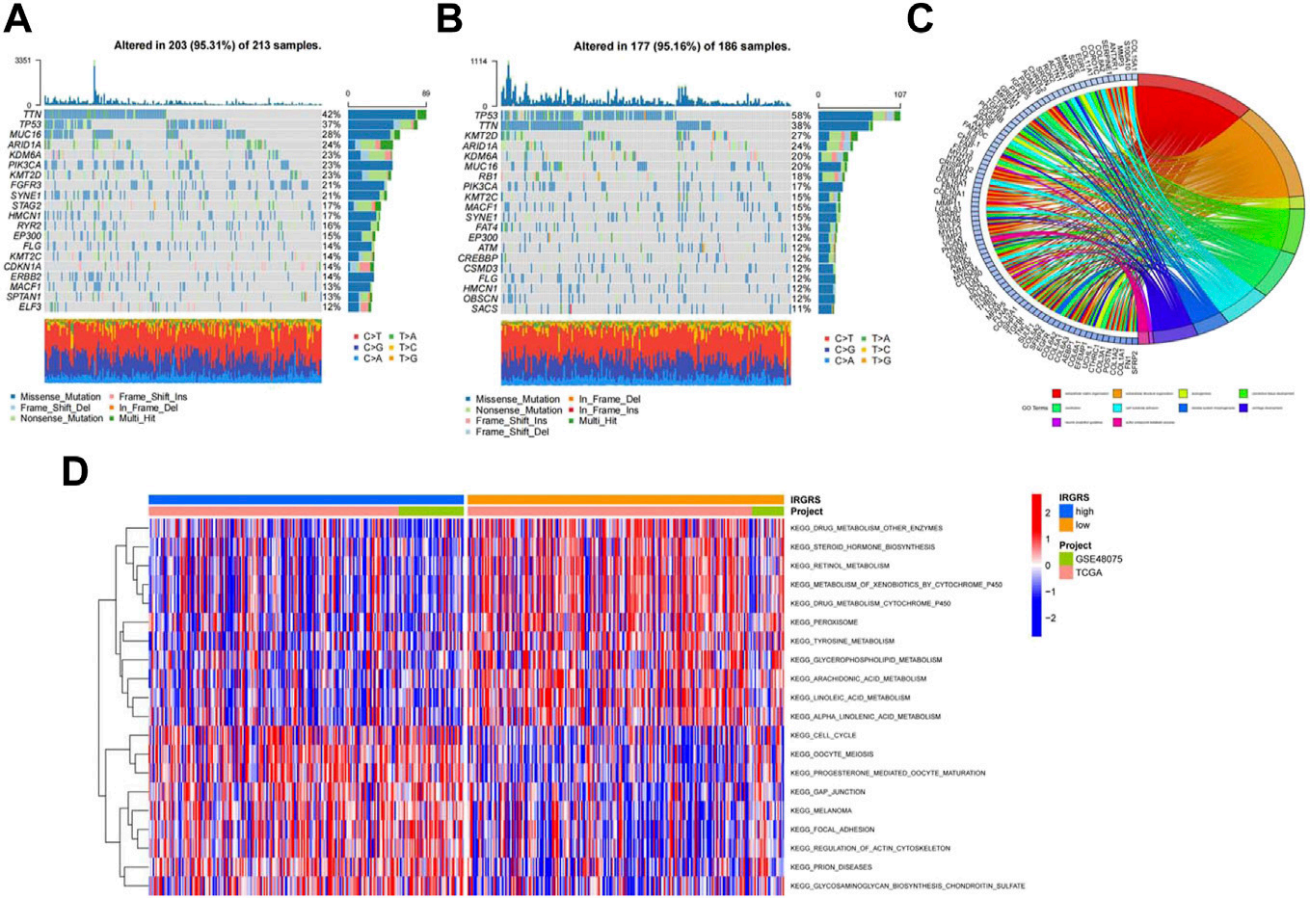
Molecular characteristic and functional enrichment analysis between high and low IRGRS groups. **(A)**. The waterfall plot of tumor somatic mutation established by those with low IRGRS groups, Mutated genes (rows, top 20) are ordered by mutation rate; Each column represented individual patients. The upper barplot showed the overall number of mutations. The right barplot showed the percentage of each variant type and the mutation frequency of each gene. The color coding indicates the mutation type. **(B)**. The waterfall plot of tumor somatic mutation established by those with high IRGRS group. **(C)**. The GO terms are defined as indicated color bars at the bottom and shown on the right of chord diagram, the involved genes are listed on the left. The genes associated ten significant signaling pathways. **(D)**. Heatmap by GSVA analysis between high and low IRGRS group.The upper barplot showed the IRGRS defined subgroups (high-IRGRS and low-IRGRS) and the origin of dataset (TCGA and IMvigor210). The rows of the heatmap showed the activation of corresponding pathways.

Then we investigated somatic mutation differences between the high- and low-IRGRS groups to further elucidate the biological mechanism of IRGRS. The two groups’mutation landscapes are depicted in Figure 4A and Figure 4B. We listed the top 20 genes with the highest mutation rates in the IRGRS subgroups and found that missense variations were the most common mutation type in both types. The mutation rates of TTN, TP53, MUC16, ARID1A, PIK3CA, KMT2D, NOTCH1, and SYNE1 were higher than 15% in both groups. The mutation rate of the TP53 and RB1 genes in the high-IRGRS subgroup was higher than that in the low-IRGRS subgroup. We determined the DEGs to further investigate the underlying biological behaviour of IRGRS by using the limma package (Ritchie et al., 2015), GO, KEGG, GSEA, GSVA, and ssGSEA were performed by The clusterProfiler package (Yu et al., 2012) and GSVA package (Huang et al., 2021) for the DEGs between the high-IRGRS group and the low-IRGRS group. We conducted GO and KEGG pathway enrichment analyses to explore the functional characteristics of the DEGs. In the GO functional enrichment analysis, the top 10 enriched biological processes were “extracellular matrix organization”, “extracellular structure organization”, “axonogenesis’, “connective tissue development”, “ossification”, “cell−substrate adhesion”, “skeletal system morphogenesis”, “cartilage development”, “neuron projection guidance” and “sulfur compound metabolic process” (Figure 4C). Based on DEGs from the IRGRS, we performed GSVA to explore the biological behaviour differences between the IRGRS subgroups. We found that the low IRGRS subgroup was markedly enriched in drug metabolism, steroid hormone biosynthesis, and retinol metabolism. The high-IRGRS subgroup presented enrichment in the cell cycle, gap junctions and regulation of the actin cytoskeleton (Figure 4D).

**FIGURE 5:**
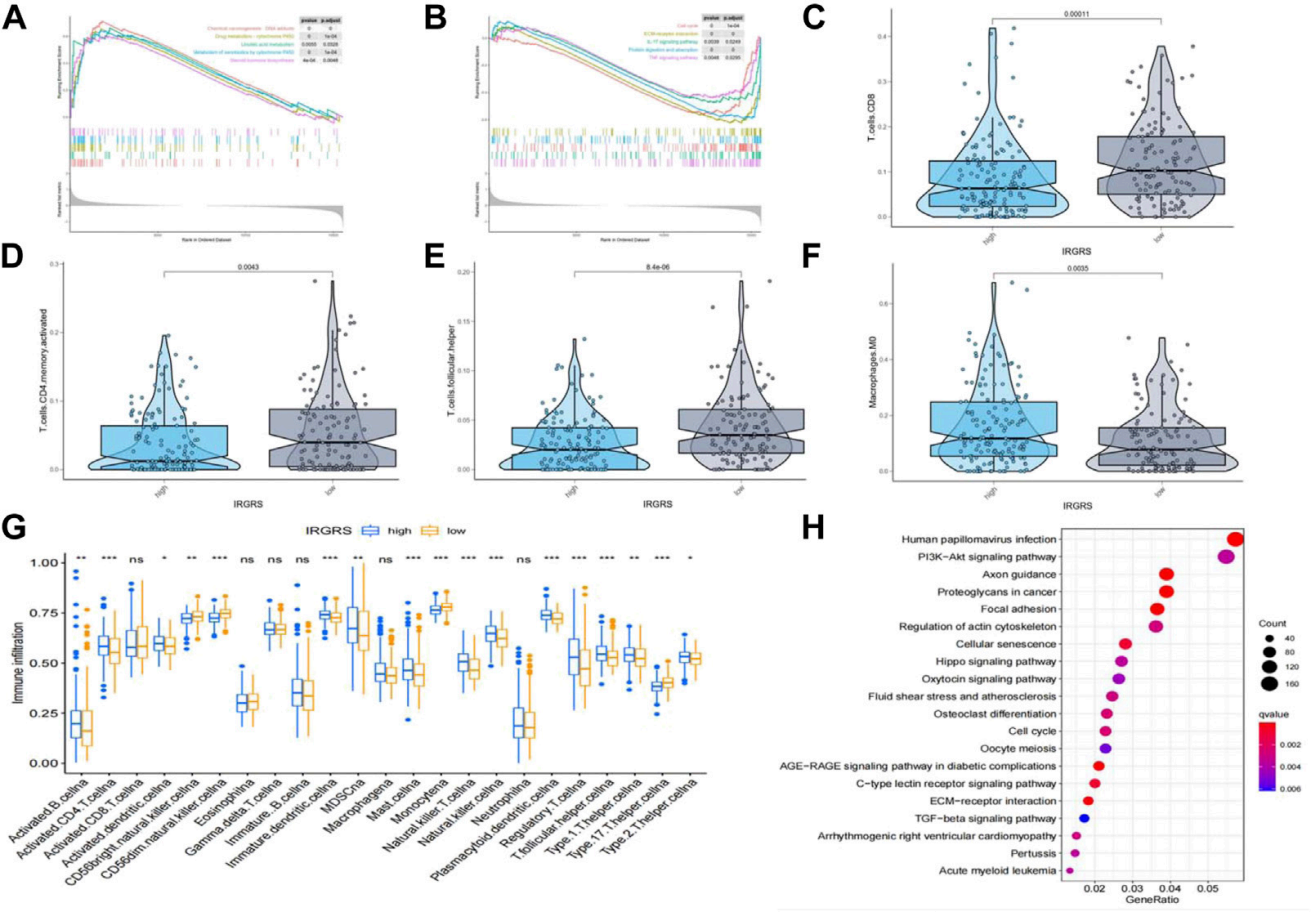
Difference in tumor infiltrated immune cells between high and low IRGRS groups. **(A)**. high score pathways enriched by GSEA analysis between high and low IRGRS groups. **(B)**. low score pathways enriched by GSEA analysis between high and low IRGRS groups. **(C–F)**. different kinds of T cells in tumor environment expressed diffrently between high and low IRGRS groups. **(G)**. tumor infiltrated immune cells analysed by ssGSEA analysis between high and low IRGRS groups. **(H)**. KEGG analysis of DEGs between high and low IRGRS groups.

We performed GSEA to identify the corresponding gene sets enriched in different IRGRS subgroups. The top five significantly enriched pathways in the high- and low-IRGRS groups are shown in Figures 5A,B. Genes in the low IRGRS groups were mostly enriched in “cell cycle”, “ECM-receptor interaction”, “IL-17 signalling pathway”, “protein digestion and absorption” and “TNF signalling pathway”. These factors are tightly associated with the immune response. Genes in the high-IRGRS group were mostly enriched in pathways related to chemical carcinogenesis and metabolism. Detailed results of the GSEA are listed in Supplementary Table S5. To evaluate how IRGRS reflects the cell type in the tumour immune microenvironment, the ESTIMATE and CIBERSORT algorithms were applied to compute the infiltration of immune cells in BLCA. Differentially infiltrated cells between the low- and high-IRGRS groups are presented in Figures 5C–F. We observed that the high-IRGRS group had lower levels of immune cells (including CD8^+^ T cell infiltration, CD4^+^ memory-activated T cells, and follicular helper T cells). Conversely, the level of M0 macrophages was higher in the high-IRGRS group than that in the low-IRGRS group. In the ssGSEA analysis(Figure 5G), many immune-related cells(including activated B cells, activited CD4^+^ T cells, immature B cell and so on) showed a higher amount in high-IRGRS group than that in low-IRGRS group. KEGG pathway analysis revealed the significant pathways between high and low IRGRS groups (Figure 5H).

### Relationship between IRGRS grouping and other immune and molecular subtypes

**FIGURE 6:**
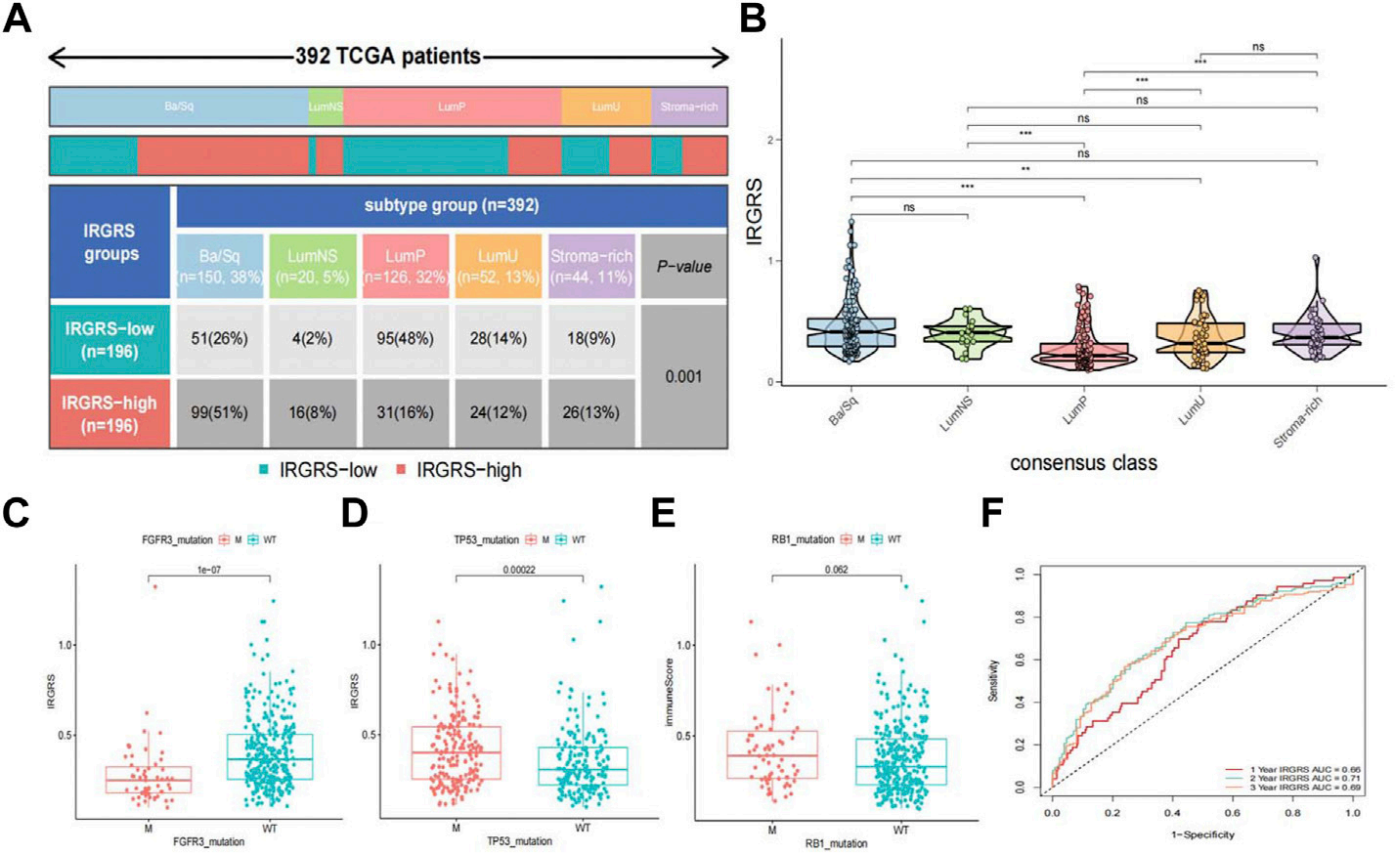
Relationship between molecular classification and IRGRS **(A)**. Heatmap and table showing the distribution of bladder cancer consensus molecular subtypes between the IRGRS and subgroups in TCGA dataset. **(B)**. Five bladder cancer molecular subtypes and their corresponding IRGRS. **(C)**. Comparison of IRGRS between the FGFR3 mutated groups and FGFR3 wild type groups.M = mutated, WT = wild type. **(D)**. Comparison of IRGRS between the TP53 mutated groups and TP53 wild type groups. **(E)** Comparison of IRGRS between the RB1 mutated groups and RB1 wild type groups. **(F)**. Time dependent ROC curve analysis of survival prediction by the IRGRS.

A consensus molecular classification subtype can describe the landscape of bladder cancer according to the RNA-sequence data and can be summarized as six molecular subtypes, namely, Ba/Sq, LumNS, LumP, LumU, stroma-rich, and NE-like (Kamoun et al., 2020), which is a classification system based on six published classification systems. Then, we focused on the distribution of different molecular subtypes in the IRGRS groups. Because the number of NE-like samples was below 10, we did not include it in our analysis. In our study, the low-IRGRS subgroup comprised 26% Ba/Sq samples, 2% LumNS samples, 48% LumP samples, 14% LumU samples, and 9% stroma-rich subtype samples, while the high-IRGRS subgroup comprised 51% Ba/Sq samples, 8% LumNS samples, 16% LumP samples, 12% LumU samples, and 13% stroma-rich subtype samples (Figure 6A). We found that the Ba/Sq and LumP subtypes accounted for a large proportion of all samples from the TCGA database. There were more LumP samples in the low-IRGRS subgroup than in the high-IRGRS subgroup (*p* < 0.01). The violin plot (Figure 6B) shows the different molecular classifications and their corresponding IRGRS. The Ba/Sq subgroup was markedly associated with a higher IRGRS than the LumP and LumU subtypes. The LumP subgroup was associated with a lower IRGRS than the LumU and stroma-rich subtypes. Several genes (such as FGFR3, TP53, and RB1) have been identified as being vital for the characterization of each consensus class (Kamoun et al., 2020). Therefore, we analysed the relationship between the IRGRS and the mutation status of the three genes mentioned (Figures 6C–E). The IRGRS of p53-mutated samples was higher than that of p53 wild-type samples (*p* < 0.01). Conversely, in the FGFR3 gene, FGFR3-mutated samples had lower IRGRS values than FGFR3 wild-type samples. However, there was no significant difference in IRGRS levels between RB1-mutated samples and wild-type samples. We evaluated the survival prediction ability of IRGRS in TCGA-BLCA datasets by using time-dependent ROC analysis. We found that the AUCs for the IRGRS were 0.66, 0.71, and 0.69 at 1, 3 and 5 years, respectively (Figure 6F).

### The benefit of immunotherapy in different IRGRS subgroups

**FIGURE 7:**
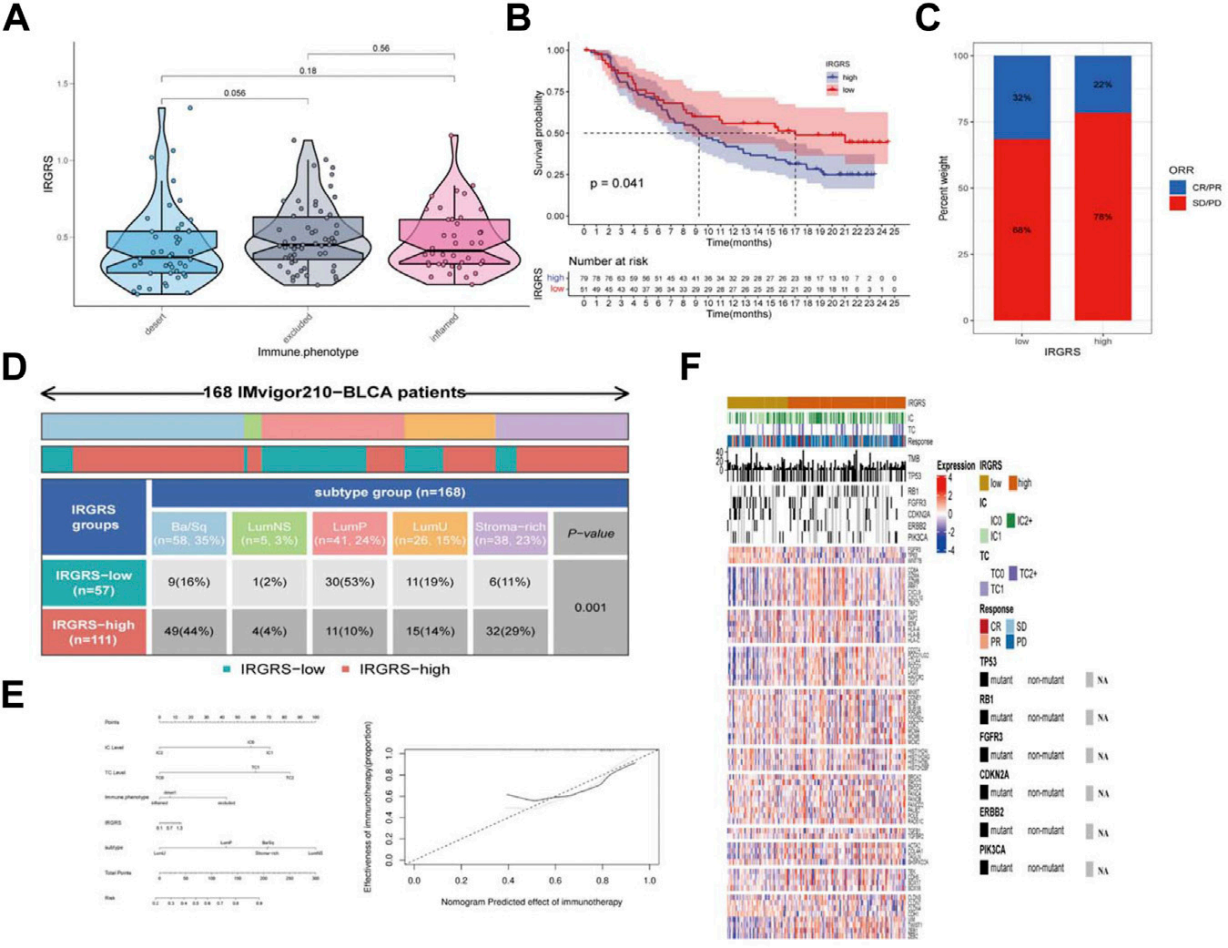
The role of IRGRS in predicting the effect of immunotherapy of bladder cancer. **(A)**. Comparison of the IRGRS of different immune phenotype in bladder cancer. **(B)**. Survival analysis of immunotherapy gene set in diferent IRGRS groups (high-IRGRS and low-IRGRS). **(C)**. The proportions of clinical response (CR/PR, SD/PD) after accepting immunotherapy in the high-IRGRS and low-IRGRS groups in IMgivor210 dataset **(D)**. Heatmap and table showing the distribution of bladder cancer consensus molecular subtypes between the IRGRS and subgroups in IMgivor210 dataset. **(E)** Nomogram and corresponding calibration curve for predicting survival probability in the validation cohort. **(F)**. Heatmap representing evaluated patients first sorted based on a IRGRS-based subtyping scheme, Immune cell and tumour cell PD-L1 status are given. Then by response to atezolizumab. In addition, TMB and mutation status (black, mutated; grey, patients without mutation data) for a few genes of interest are shown. The rows of the heatmap show expression (Z scores) of genes of interest, grouped into the biologies or pathways.

We then explored the potential clinical efficacy of IRGRS in predicting the effect of immunotherapy based on the IMvigor210 dataset. All samples were classified into immune-desert, immune-excluded and immune-inflamed phenotypes. The immune-desert phenotype was characterized by the suppression of immunity. The immune-excluded phenotype was characterized by innate immune cell infiltration and stromal activation. The immune-inflamed phenotype was characterized by adaptive immune cell infiltration and immune activation. In our results, the immune-excluded phenotype had a higher IRGRS than those of the other two subgroups, implying that high-IRGRS patients could benefit less from immunotherapy than low-IRGRS patients (Figure 7A). Then, we evaluated whether the IRGRS could predict patients’ response to immune checkpoint blockade therapy based on IMvigor210 cohorts. Survival analysis (Figure 7B) showed that high-IRGRS patients had worse OS than low-IRGRS patients, which was consistent with the results of the training datasets. We included Ba/Sq, LumNS, LumP, LumU four subgroups into our survival analysis. Patients with a low IRGRS exhibited a greater clinical response to anti-PD-1/L1 immunotherapy than those with a high IRGRS (Figure 7C). We could find from Figure 7D that there were more Ba/Sq samples and fewer Lump samples in the high-IRGRS subgroup than in the low-IRGRS subgroup (*p* < 0.001, x2 test). The result of which was consistent with training dataset from TCGA + GSE48075. Given that the immune cell(IC) level, tumor cell(TC) level, immune phenotype, consensus subtype had been shown to be highly predictive of the response to immune therapy (Tsao et al., 2018; Kamoun et al., 2020; Hornburg et al., 2021), we speculated that they might function as synergistic factors in predicting the response to immunotherapy. Therefore, a nomogram was developed to include all factors above to offer a quantitative approach for predicting the effect of immunotherapy. The nomogram was constructed in the IMvigor210 cohort and the corresponding calibration curve was constructed (Figure 7E). To find a relationship between IRGRS and bladder cancer immune landscape, we portray the IRGRS-defined subgroups to contrast the IRGRS-defined subgroups with IC, TC and immunotherapy responsein (Figure 7F). Consistent with the importance of TMB, we observed that the low-IRGRS subtype was significantly enriched for response of immunotherapy.

## Discussion

Increasing evidence suggests that the tumour immune microenvironment plays an important role in innate immunity as well as antitumour effects through interactions between immune cells and tumour cells (Binnewies et al., 2018). Based on the mechanism of immune evasion, immunotherapy has proven to be effective for patients unsuitable or recurrent after cisplatin-based treatment. However, only a few patients can benefit from immunotherapy (Ott et al., 2020). Biomarkers including PD-1 expression, PD-L1 expression, tumour mutation burden and MSI status are not efficient for predicting the benefits of immune checkpoint blockade (Subrahmanyam et al., 2018; Ganesh et al., 2019; Jardim et al., 2021). In addition, the clinical prognosis heterogeneity of BLCA reveals that immune-relevant subtypes may exist between BLCA samples in the same clinical stage. This situation highlights the urgent need to develop a robust biomarker and subgroup analysis for guiding immunotherapy in BLCA.

In our study, based on 22 tumour-infiltrated immune cell lines, we identified four distinct tumour microenvironment patterns. These four patterns had significantly different tumour-related immune cell characteristics. Cluster A was characterized by a low expression level of CD8^+^ T cells and a high level of resting memory CD4^+^ T cells. In contrast, cluster B displayed more CD8^+^ T cells and less resting memory CD4^+^ T cells. Cluster C showed a higher resting mast cell quantity than those of the other clusters. Cluster D was characterized by a high level of M0 macrophages. Each TME cluster showed unique features with respect to the tumour-infiltrated immune microenvironment. In many previous studies, only the results from the transcriptome profile and enriched pathways associated with immunity were considered. However, in our study, to identify the underlying mechanism and hub genes connected with the TME clusters, we conducted several computational algorithms to construct an IRGRS system. The IRGRS is proven to be a robust biomarker for guiding the immunotherapy of bladder cancer, with better survival in low-IRGRS patients and worse survival in high-IRGRS patients in both training and validation cohorts.

The IRGRS consists of three genes: EGFR, OAS1, and MST1R. Epidermal growth factor receptor (EGFR) is widely recognized because it is of great importance in many kinds of cancers (Ganesh et al., 2019; Zeng et al., 2020). Mutations and amplification in its exon region have been identified to be driving events in many cancer types. The protein encoded by EGFR is a receptor for members of the epidermal growth factor family. Many research and drug development efforts have been prompted by its role in non-small-cell lung cancer (Harrison et al., 2020), basal-like breast cancers (Gonzalez-Conchas et al., 2018) and glioblastoma (Eskilsson et al., 2018). Tyrosine kinase inhibitors such as gefitinib and erlotinib have shown efficacy in tumours with EGFR exon amplification. However, some studies revealed that patients diagnosed with EGFR-mutated non-small-cell lung cancer could draw limited benefit from immunotherapy (Proto et al., 2019). These results suggest that the EGFR gene is a vital factor influencing whether immunotherapy can exert a positive effect in patients. In addition, EGFR has been identified as an oncogenetic mechanism in the basal/squamous (Ba/Sq) subtype among the six molecular classification subtypes (Kamoun et al., 2020). Oligodenylated synthase 1 (OAS1) is a protein encoded by OAS1 that results in RNA degradation and the inhibition of viral replication; it has been included in several prognostic signatures and has been found to be a robust biomarker to predict the effect of immunotherapy (Luo et al., 2020; Jin et al., 2021). Macrophage stimulating 1 receptor (MST1R) is a gene that encodes a cell surface receptor for macrophage-stimulating protein with tyrosine kinase activity. Studies have found that suppression of MST1R expression results in reduced pancreatic tumour size, changes in macrophage polarization and enhanced T-cell infiltration (Braun et al., 2018; Tan et al., 2019).

Then, we studied the gene mutations of different IRGRS subgroups to uncover the underlying immunologic mechanism. The most common gene mutations in both the high-IRGRS and low-IRGRS samples were missense variations. However, for some other mutation types, such as nonsense mutations and frameshift mutations, there was quite a difference between the different IRGRS groups. TP53 mutation was the most differentially expressed gene in the top 20 mutated genes between high-IRGRS and low-IRGRS samples. TP53 mutation is one the most common mutation types in many kinds of cancer and can lead to poor outcomes (Vousden and Prives, 2005; Olivier et al., 2006). TP53 can regulate the p53/TGFβ signalling pathway, which has an influence on tumour cell proliferation by the cell cycle. In addition, there was a higher rate of RB1 mutation in the high-IRGRS subgroup than in the low-IRGRS subgroup. RB1 was the first tumour suppressor gene found, and the protein encoded by RB1 is a negative regulator of the cell cycle. Therefore, high-IRGRS patients with high TP53 and RB1 mutation burdens have a worse outcome than low-IRGRS patients with low TP53 and RB1 mutation burdens, in agreement with our survival results. GO, GSVA, and GSEA analyses between the high and low IRGRS groups suggest that apart from activated immune-related pathways, there are also many other mechanisms in the tumour immune microenvironment.

Several molecular classifications have been reported in the development of a more precise patient stratification (Choi et al., 2014; Robertson et al., 2018; Tan et al., 2019). However, even the consensus classification system of subtypes has not translated universally into clinical trials or clinical applications (Kamoun et al., 2020). Thus, we analysed the association between the IRGRS and the consensus classification system (LumP, LumU, stroma-rich, LumNS, and Ba/Sq). Each consensus class has distinct differentiation patterns, oncogenic mechanisms, tumour microenvironments, and histological and clinical associations.For example, the tumor driving mechanism of the Lump subtype is mainly related to the overexpression of FGFR3, and the Ba/Sq subtype is mainly related to the overexpression of EGFR.In addition, the mutation spectrum of different molecular subtypes is also different. For example, the mutation rate of the RB1 gene in the Ba/Sq subtype is significantly higher than that of other subtypes, and the KDM6A gene has the highest mutation rate in the Lump subtype. We found that more than half of the high-IRGRS samples were distributed in the Ba/Sq classification, and nearly half of the low-IRGRS samples were enriched in the LumP classification. The Ba/Sq subtype was identified to be more sensitive to immunotherapy than the other subtypes (Kamoun et al., 2020), which was consistent with our results. FGFR3 mutation has been recognized as one of the oncogenic mechanisms in the development of the LumP subtype of MIBC (Robertson et al., 2017). We also revealed that the IRGRS correlated with FGFR3 mutation in our study. FGFR3-targeted therapy may be an encouraging choice for low-IRGRS tumours, especially in the LumP subtype of MIBC. Molecular classification of bladder cancer showed tumour biological heterogeneity, which could provide an innovative approach to improving therapeutic effectiveness. When we combined the IRGRS with a molecular classification system, we could classify MIBC subgroups and guide personalized antitumour therapies more precisely. Prospective clinical trials need to be performed to certify the therapy-related predictive value of the IRGRS, and certain therapies need more investigation through *in vitro* or *in vivo* experiments.

Then, we confirmed the effect of the IRGRS in predicting the efficacy of immunotherapy based on the IMvigor210 dataset. We found different immune microenvironment-related cells between the high and low IRGRS groups, which might partly explain the different responses to immunotherapy between the two groups. Integration with 3 immune-related subtypes (immune desert, immune exclusion, and immune inflamed) allowed IRGRS grouping to distinguish different immune subtypes of BLCA. Unfortunately, there were no significant differences between the immune-desert and immune-excluded groups, which may be because the number of samples in the IMvigor210 dataset was not large enough. It has been recognized that the effective rate of immunotherapy for PD-L1 positive bladder cancer patients is only about 20%, which suggests the limitation of PD-L1 as an indicator. While in patients with low IRGRS, the effectiveness of immunotherapy can reach 32%. This further demonstrates the superiority of the IRGRS. More importantly, our study has developed several new insights for bladder cancer immunotherapy that target the IRGRS phenotype and immune phenotype. By combining the IRGRS and molecular classification, we might select patients who are suitable for immunotherapy more accurately. Further reversing the adverse TME cell infiltration may contribute to exploiting the development of novel drug combination strategies or novel immunotherapeutic agents in the future. Moreover, the patients in the high-IRGRS group had a shorter follow-up time than those in the low-IRGRS group. Several limitations of this study should be considered. First, Recent studies suggested that OICR-9429 and HSF1 played important roles in regulating the tumor microenvironment of bladder cancer. They conducted in-depth study about how the two genes work (Zhang et al., 2021b; Huang et al., 2022). Although we have reviewed the roles of the three genes that construct IRGRS in tumors, the underlying molecular mechanisms require further exploration of *in vivo* and *in vitro* functional experiments. Second, our study is a bioinformatics analysis based on public databases and lacks validation of independent clinical cohorts.

## Conclusion

By applying a series of bioinformatics methods, we constructed IRGRS that could accurately predict the effect of immunotherapy and prognosis in bladder cancer. In addition, when we conbined IRGRS with bladder cancer consensus classification system, we could improve the robustness of prediction. However, further prospective clinical studies are needed to verify the absoluteness of our conclusion.

## Data Availability

All the data used in this study are from public datasets (TCGA, GEO, IMvigor210) and can be accessed without restriction.

## Abbreviations

AUC, Area under curve; BLCA, Bladder cancer; CDF, Cumulative distribution function; CIBERSORT, Cell-type Identification by Estimating Relative Subsets of RNA Transcripts; DEGs, Diferentially expressed genes; GSEA, Gene set enrichment analysis; GSVA, Gene set variation analysis; GO, Gene oncology; GEO, Gene Expression Omnibus; GS, Gene significance; IRGRS, Immune related gene risk score; KEGG, Kyoto Encyclopedia of Genes and Genomes; LASSO, Least absolute shrinkage and selection operator; MM, Module membership; OS, Overall survival; ROC, Area under the curve; ssGSEA, single sample gene set enrichment analysis; TMB, Tumour mutation burden; TCGA, The Cancer Genome Atlas; TPM, Transcripts per million; TOM, Topological overlap matrix; WGCNA, Weighted gene coexpression network analysis.
