# Supplementary figures and images for: Immune-related gene risk score predicting the effect of immunotherapy and prognosis in bladder cancer patients
Source: Front Genet. 2022 Oct 4;13:1011390. doi: 10.3389/fgene.2022.1011390 (PMC9577248; doi:10.3389/fgene.2022.1011390)

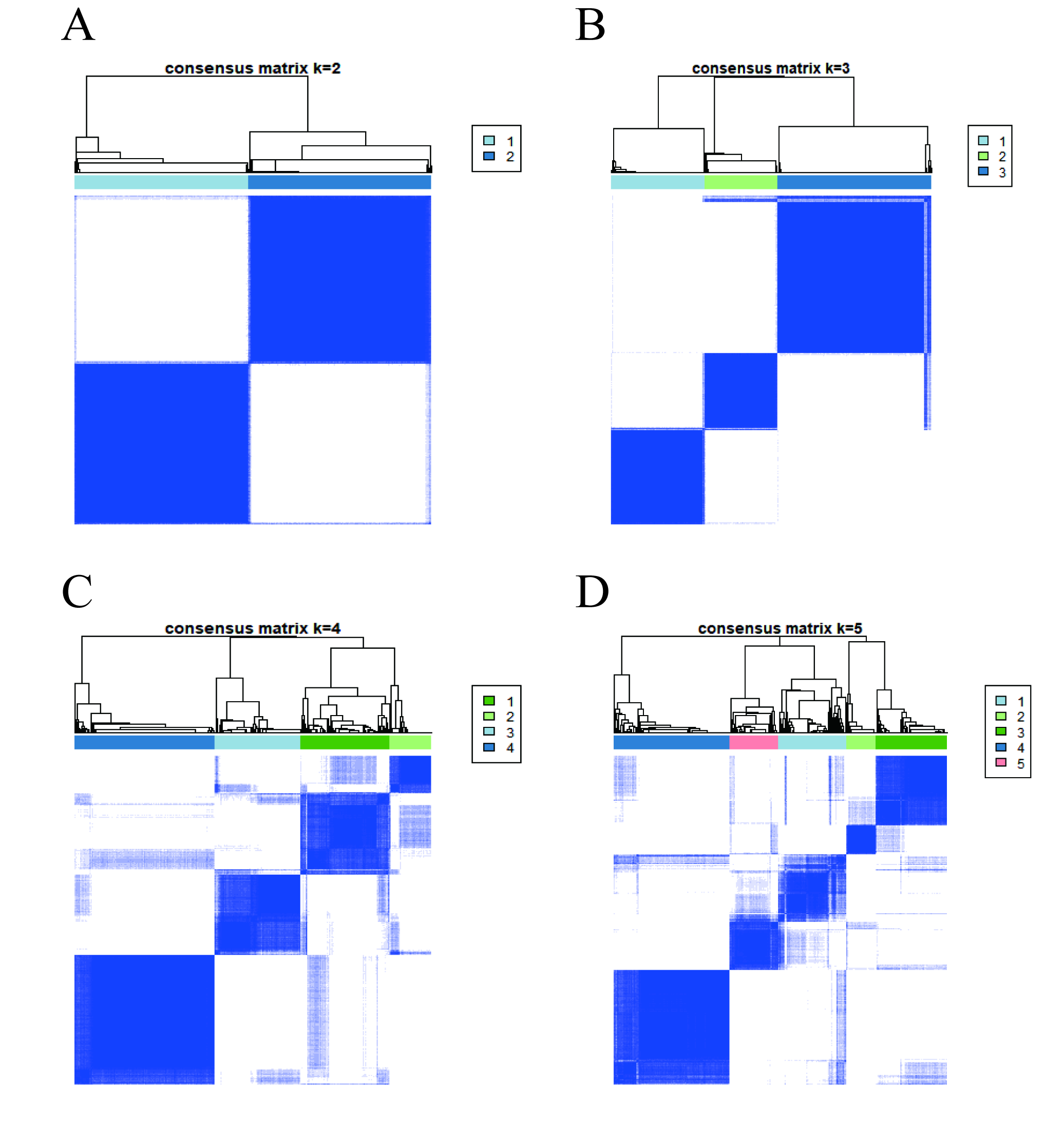

Supplement: Supplementary file 5 [file Image2.TIF]

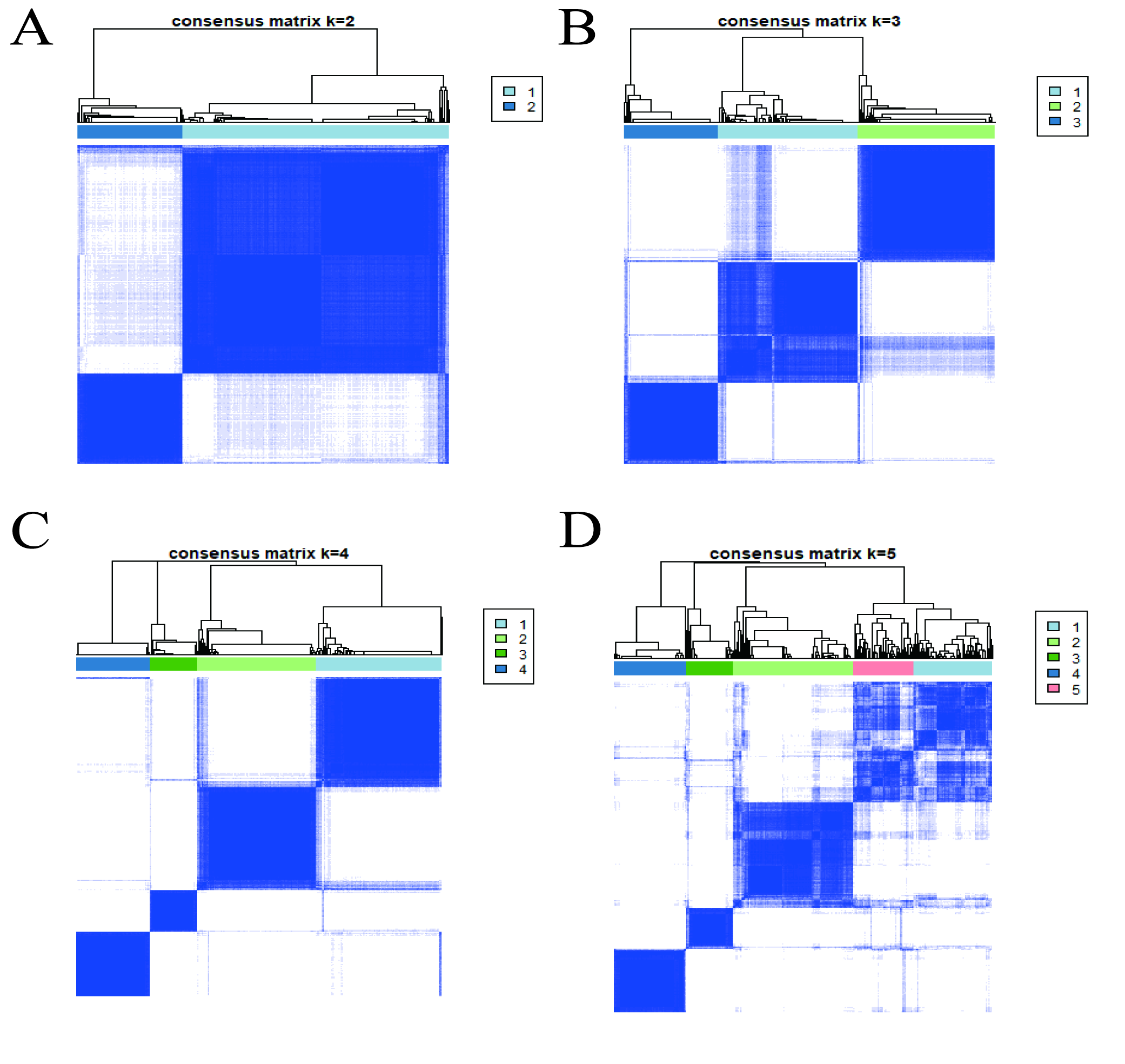

Supplement: Supplementary file 7 [file Image1.TIF]
